# GlycoViz: a glycoproteomics tool for validating and visualizing glycopeptide identifications

**DOI:** 10.1093/bioinformatics/btag691

**Published:** 2026-09-21

**Authors:** Wenzhou Li, Niclas Olsson, Fiona E McAllister

**Affiliations:** Calico Life Sciences LLC, South San Francisco, CA 94080, United States; Calico Life Sciences LLC, South San Francisco, CA 94080, United States; Calico Life Sciences LLC, South San Francisco, CA 94080, United States

## Abstract

**Summary:**

GlycoViz is an open-source algorithm and software tool designed to address critical bottlenecks in the post-identification workflow of mass spectrometry-based glycoproteomics. Current glyco search engines excel at glycopeptide identification but there is a lack of tools for robust post-identification validation, global glycan composition statistics, and reliable site-specific analysis, particularly concerning false positives and quantification distortion due to shallow detection depth. GlycoViz offers an alternative solution with a novel glycopeptide validation algorithm that is compatible with data from multiple search engines. Analysis of N-linked glycans is fully supported whereas O-linked glycan data analysis is limited primarily to visualization and non-optimized validation.

**Availability:**

The open-source software is available on GitHub at https://github.com/calico/glycoviz. It can be launched as local software through Docker Compose or hosted as a web application in a server. A snapshot of the code is available on Zenodo: https://doi.org/10.5281/zenodo.22680123

## 1 Introduction

Glycosylation of proteins is one of the most common post translational modifications. Measuring glycosylation of proteins ranges from the analysis of a single protein (e.g. recombinant monoclonal antibody) to global glycoproteome analysis of the entire proteome in plasma, cells, and tissues. Different analysis workflows have been developed for these two extremes of the spectrum.

First, glycoproteomics, the large-scale study of protein glycosylation, is critical for understanding numerous biological processes, disease mechanisms, and therapeutic targets. Mass spectrometry-based glycopeptide identification has advanced significantly through specific search engines like MSFragger (Polasky *et al.* 2020) and Byonic (Bern *et al.* 2012), but a significant bottleneck remains in the post-identification workflow. Current tools primarily focus on *identifying* glycopeptides. Glycoproteomics researchers then typically use in-house scripts to perform global glycan composition statistics to provide insights into the glycan differences between samples (Riley *et al.* 2019, Fang *et al.* 2025, Wu *et al.* 2026). This approach rarely extends to the analysis of a specific protein or glycosite in the dataset because most sites have incomplete identification profiles, thus making it difficult to validate whether a glycan from a particular site is a misidentification or whether the quantitation is distorted due to shallow detection depth of different glycan species. A recent study comparing glyco search engines found that approximately half of the identified glycopeptides were unique to individual tools (Sakaue *et al.* 2025), highlighting the necessity to manually validate the authenticity of individual glycan IDs for any protein of interest.

Second, glycan profiling of purified protein drugs is a standard, well-established practice in the pharmaceutical industry, typically utilizing methods such as LC-MS/MS based peptide mapping and Hydrophilic Interaction Liquid Chromatography (HILIC) mapping of released glycans (Zhang *et al.* 2016). These assays rely on the comprehensive identification of glycan species at each site and subsequent peak area-based quantitation to determine the percentage of each glycan species. While these analysis methods are robust, reliable, and routinely employed during the process development of drug substances and products, their application to glycoproteomics is limited. The primary challenge is the difficulty in obtaining a deep glycan profile for most sites within a complex proteome.

Beyond upstream identification and quantitative workflows, attention has increasingly shifted toward downstream post-processing and visualization tools. For example, GlyConnect (Alocci *et al.* 2019) maps literature-curated relationships between glycoproteins, specific glycosites, and glycan structures; GlycoWork (Thomès *et al.* 2021) leverages deep learning to analyze structural glycan motifs; GlycoDiveR (Veth and Riley 2026) standardizes and visualizes high-dimensional glycopeptide datasets through a modular R framework. While highly valuable, these platforms are primarily exploratory or reference-based. Crucially, they assume that upstream search-engine outputs are entirely flawless, leaving researchers to manage the high false-positive rates downstream.

To address these limitations, we developed GlycoViz, a search-engine-agnostic tool designed to bridge three traditionally isolated analytical pain points:

- *Global Glycan Statistics:* GlycoViz automates macro-level profiling across entire cohorts, allowing researchers to capture broad glycosylation trends without requiring advanced bioinformatics or scripting skills.
- *Protein-Centric Analysis:* Rather than treating data as flat list outputs, GlycoViz enables researchers to easily zoom in on specific target proteins to map their microheterogeneity and trace how individual glycoforms behave under different experimental conditions.
- *Site-Level Validation:* Uniquely, GlycoViz goes beyond exploratory plotting by addressing the critical issue of data reliability. It features a novel, built-in validation algorithm that automatically calculates the analytical confidence of individual glycosites, filtering out true versus false positives. By dynamically evaluating site-level identification and quantification, GlycoViz drastically reduces the bottleneck of manual spectrum validation.

Currently, GlycoViz is optimized primarily for N-linked glycans. Support for O-linked glycans is less advanced. Basic data processing and visualization are supported, standardized nomenclature and the automated validation algorithm have not yet been validated for O-linked datasets.

## 2 Methods

### 2.1 Validation algorithm

GlycoViz enables the validation of glycans based on several orthogonal criteria in addition to the traditional search engine scores. GlycoViz creates a composite score using a rank-percentile approach, which effectively normalizes metrics of different scales and minimizes the influence of outliers. Each contributing metric is transformed such that it ranges from 0 to 1, and the direction is oriented so that a higher value always indicates higher confidence.

Orthogonal criteria used includes:

- **Detection depth:** A measure of the number of times a glycopeptide was identified globally across multiple experimental runs or replicates, normalized by the number of glycan species identified for a particular site. This criterion is based on the fact that glycan species are highly organism dependent, forming a unique “glycan signature” or “identity card” for different cells, tissues, and organisms. Lower rank in depth (i.e. rarer glycan species) correlates with lower confidence. This criterion is particularly useful for low coverage glycan sites.
- **Retention time conflict**: Quantifies the deviation of an observed glycopeptide retention time (RT) from a predicted or expected RT based on its peptide sequence and glycan composition. Though predicting retention time of glycopeptides with different peptide sequences is challenging, the predictions of glyco peptide RT for the same sequence are usually very accurate. Recently MSFragger published a match-between-glycans algorithm to utilize unit shift of glycan composition followed by LDA to predict glycan retention time shift (Shen *et al.* 2026). The glycoViz algorithm utilizes a similar unit shift method but is based on pairwise retention-time ordering conflicts for each glycan at a site to evaluate the agreement of experimental RT ordering with predicted ordering. Specifically, for each glycan *i* at a site, it compares against every other glycan *j* at the same site. A conflict occurs when the experimental RT difference and the predicted RT difference point are in opposite directions. Though the model does not calculate the magnitude of disagreement, it is more robust to inaccuracies in RT and sites with fewer glycans.
- **Search engine score**: Score from search engine which reflects confidence for identification based on MS2.

A combined score is provided as a weighted sum of these normalized metrics:


$$\mathrm{Score}=\sum \;w_{i}\cdot \;\left(1-\mathrm{Percentile}\left(\mathrm{Metri}c_{i}\right)\right)$$

By default, the weights of each metric are pre-set. However, the platform allows users to tune these weights based on specific domain knowledge or experimental design to prioritize certain validation criteria. These selected criteria mimic the rigorous manual validation protocols standard in biopharmaceutical development, where glycan profile consistency, chromatographic retention order, and MS2 spectral quality are systematically scrutinized to distinguish true biological signals from false positives. Although the composite score is a convenient summary for ranking hits, users should inspect individual component scores to accurately interpret the source of an identification’s confidence.

Since the algorithm operates at the level of individual glycosylation sites, validation against a protein with a well-characterized glycan profile provides the most meaningful benchmark. We applied the algorithm to the NIST monoclonal antibody reference material 8671 (NISTmab), whose established glycoform profile serves as a definitive ground truth (Hilliard *et al.* 2017, Remoroza *et al.* 2024). The scoring details and the comparison with ground truth are included in the supplementary data 3. In short, the five false positives ranked 10th and 13th to 16th among 16 identified glycans. This yields an AUC (Area Under the ROC Curve) of 0.96, demonstrating the algorithm’s strong ability to distinguish true glycan identifications from false positives. As an example, we include a mouse plasma glycoproteomics dataset, searched with Byonic, to demonstrate the algorithm and software. Samples were collected from two cohorts representing different age groups. Figure 1 presents a representative glycosite plot generated by the software.

**Figure 1:**
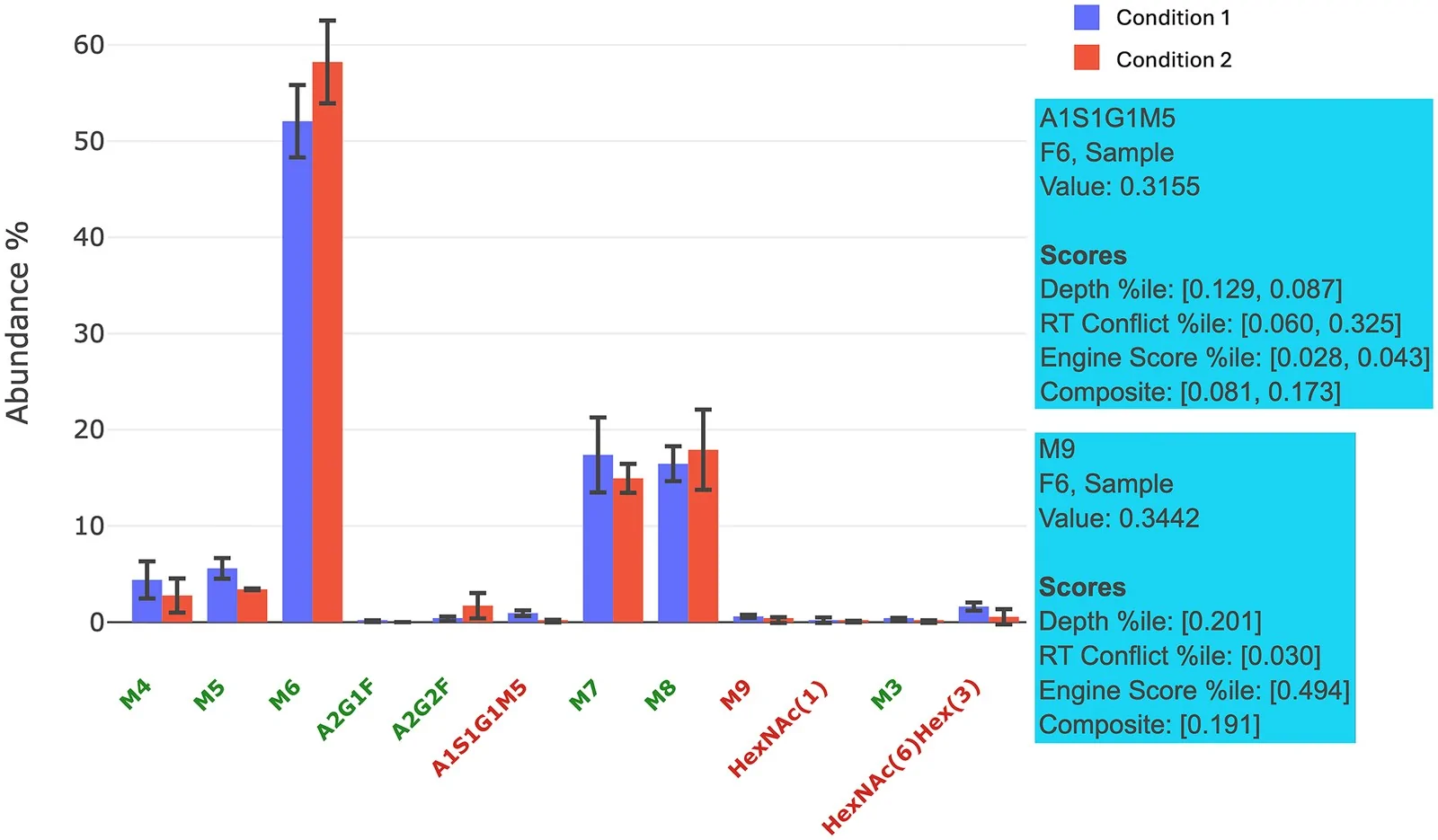
Representative glycosite plot. Colored labels denote the identification confidence as assessed by the validation algorithm for mouse IgM (A0A075B5P6) N442 site. In the interactive software interface, hovering over the data points displays the detailed component scores (blue boxes).

### 2.2 Implementations

GlycoViz is specifically designed to transition raw glycopeptide identification lists into high-confidence, biologically meaningful results. Its core functionalities are centered on rigorous data validation and insightful visualization. The software is implemented with a modern framework including a React frontend, a FastAPI backend, and a PostgreSQL database. Detailed technology stack and version requirements are documented in the “Architecture” section of the repository’s README.md file. Software implementation was assisted by the LLM-powered AI coding tool Claude Code (Opus 4.6, Anthropic). The application can be launched locally with a single “docker compose up” command or hosted on a server for organizational use. The application does not rely on external APIs with the exception of querying UniProt for known glycan sites.

GlycoViz provides an intuitive suite of features for data exploration, enabling researchers to quickly extract biological insights that are often obscured in raw search engine output. The table below summarizes the main features of the software.

**Table:** 

Feature	Description	Biological Insight
**Nomenclature conversion**	Convert glycan composition to structure-based Oxford nomenclature (Harvey *et al.* 2009) which is widely adopted in the pharmaceutical industry, e.g. BioPharma Finder software (Thermo Fisher Scientific, Waltham, MA, USA).	Bridge the gap between glycan names and align with practices in the pharmaceutical industry. e.g. HexNAc(4)Hex(4)NeuAc (1)Fuc(1) is converted to “A2S1G1F”, denoting a bi-antennary (A2) structure with one sialic acid (S1), one galactose (G1), and core fucose (F).
**Global glycan profiling statistics**	Interactive plots (e.g. pie charts, stacked bar charts) showing the distribution of glycosites and modalities across the entire dataset.	Understand the overall glycoproteomic profile and complexity.
**Differential analysis**	Volcano plot showing the glyco changes across samples and conditions.	Discover differentially expressed glycopeptides.
**Glycosite mapping**	Group glycan identifications based on sites, applying validation algorithms to indicate reliability.	Investigate changes for a particular glycosite, or combine a group of sites of interest.
**Protein-centric view**	Summarizes all identified glycan sites, species and their relative occupancy for a specific protein of interest.	Analyze microheterogeneity at the single protein level, and provide a comparison with reported glycosites from UniProt.
**Heatmap**	Cluster samples or identified glyco peptides to identify similarity and trends between samples and glycans.	Identify groups of related samples or glycan structures.
**Compare two analyses**	Perform a non-quantitative comparison of two saved analyses	Evaluate differences between proteins and glycans identified from datasets, search engines, and different parameters.

GlycoViz accepts standard tabular output files from common search engines used for glycoproteomics including MSFragger and Byonic. It also provides a customizable interface allowing users to define custom input formats for other search engine outputs. The software is available on GitHub at https://github.com/calico/glycoviz. All user data is stored locally on the computer hosting the application.

## 3 Conclusion

In summary, GlycoViz fills a critical gap in mass spectrometry-based glycoproteomics by seamlessly bridging search-engine outputs with automated site-level validation and multi-scale visualization. By replacing fragmented custom scripts and labor-intensive manual inspection with a standardized, search-engine-agnostic platform, GlycoViz enables researchers to smoothly transition from cohort-wide composition trends down to individual protein microheterogeneity.

## Supplementary Material

btag691_Supplementary_Data
