# Supplementary material for: GlycoViz: a glycoproteomics tool for validating and visualizing glycopeptide identifications
Source: Bioinformatics. 2026 Sep 21;42(10):btag691. doi: 10.1093/bioinformatics/btag691 (PMC13633667; doi:10.1093/bioinformatics/btag691)
Supplement: Supplementary file 1 [file btag691_supplementary_data.pdf]

SUPPLEMENTARY DATA for GlycoViz:

Q3TJ94 – N144 (by Replicate)

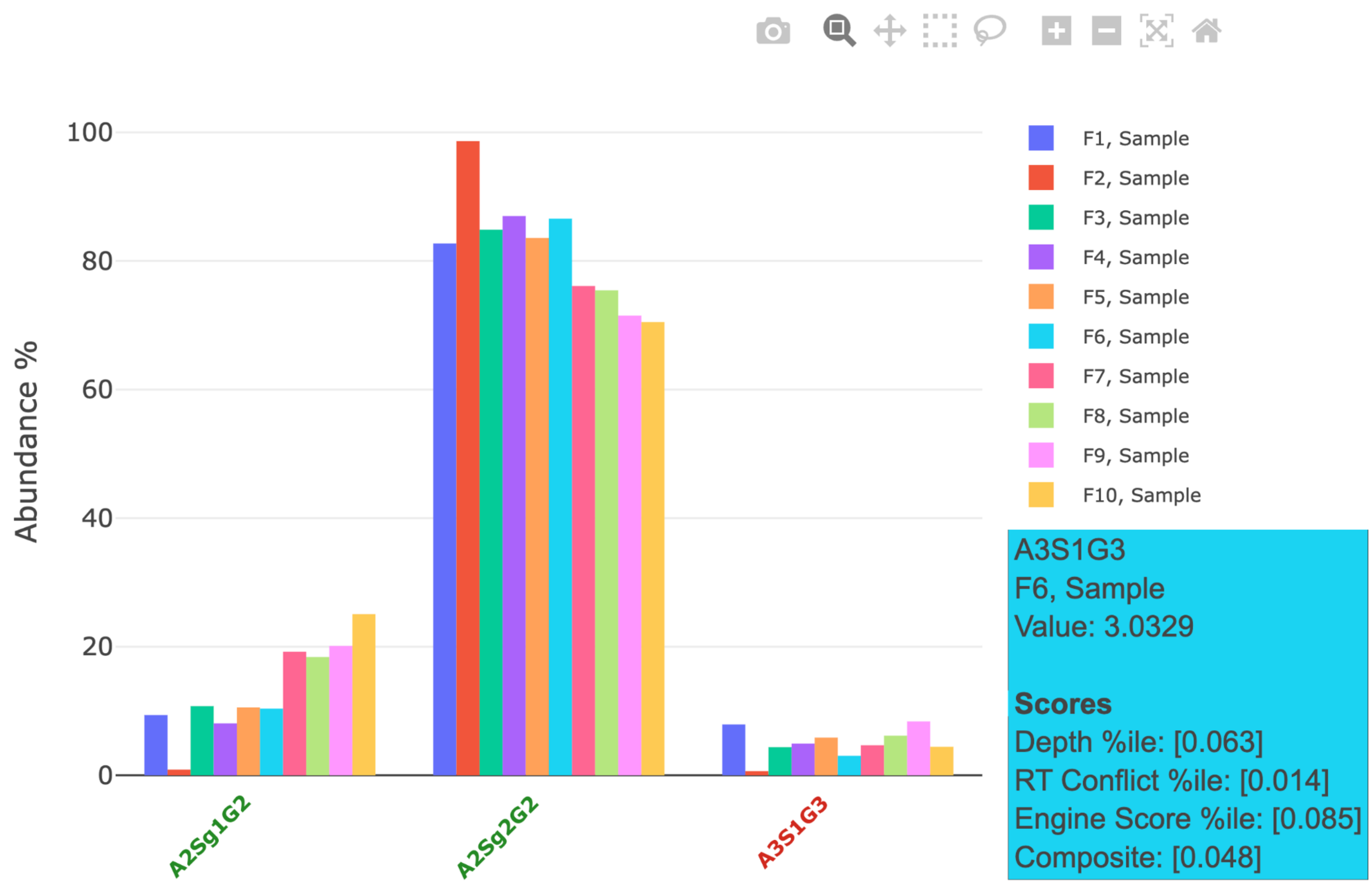

Supplement Figure 1: An example of a glycosite with limited glycan type coverage. The identification of A3S1G3 is assigned low confidence due to the poor detection depth, RT score and search engine score. It correlates well with the low abundance observed in the plot, and is consistent with the established knowledge that triantennary (A3) and N-acetylneuraminic acid (S) are rare in mice. Note: In the software interface, hovering over the plot displays a detailed score box (blue).

A0A075B5P6 – N442 (by Condition)

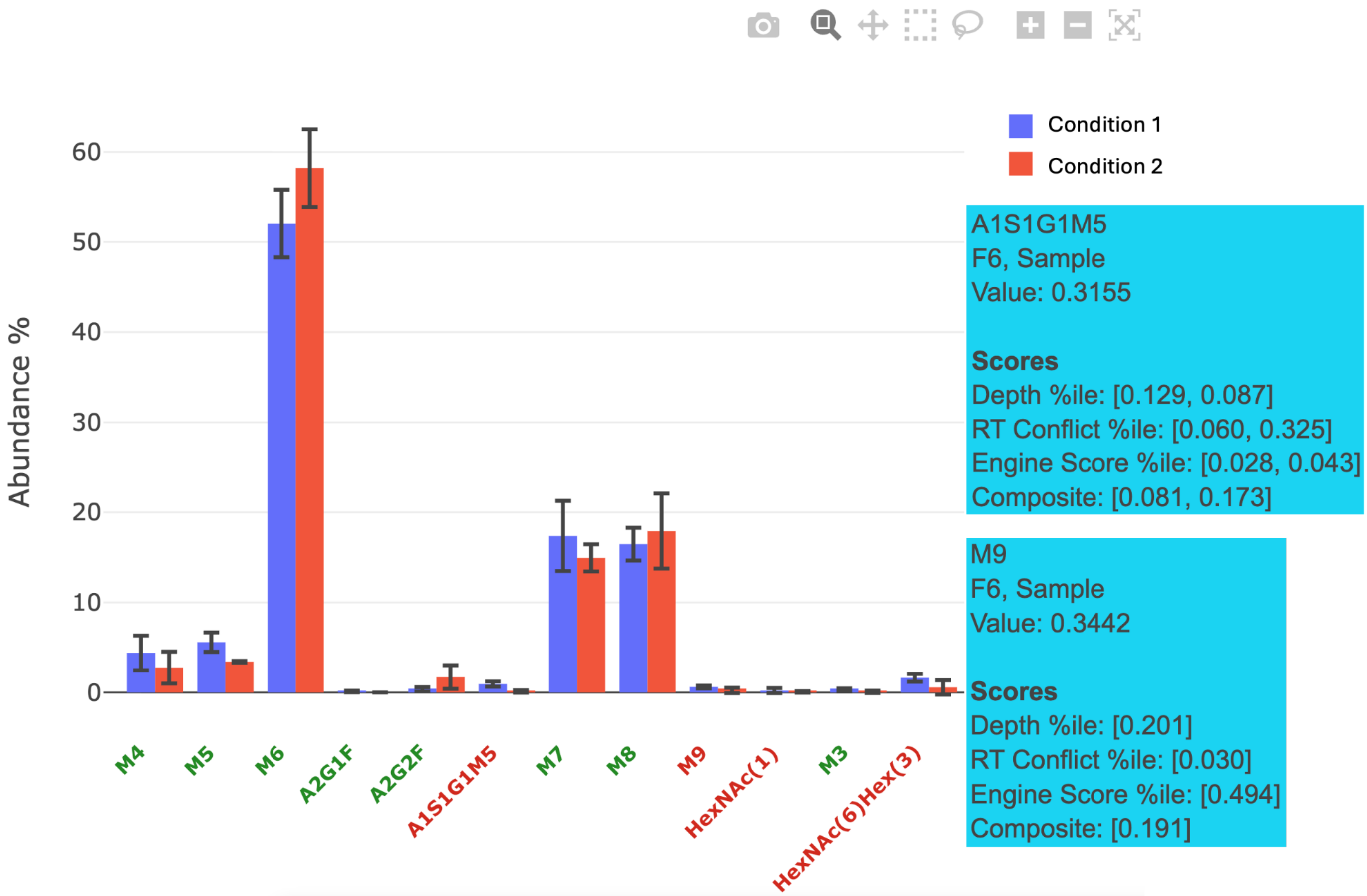

Supplement Figure 2: Comprehensive glycan type coverage at a representative mouse IgM glycosite.. M9 is labeled as low confidence due to its poor RT conflict score. HexNAc(1) and HexNAc(6)Hex(3) are likely in-source fragmentation products from other glycans. A1S1G1M5 has a lower score across all metrics. This identification is considered a probable false positive, given that mouse immunoglobulin sialylation is primarily N-glycolylneuraminic acid (Neu5Gc, Sg) rather than N-acetylneuraminic acid (Neu5Ac, S) . Reference : Krištić, J., Zaytseva, O. O., Ram, R., Nguyen, Q., Novokmet, M., Vučković, F., ... & Lauc, G. (2018). Profiling and genetic control of the murine immunoglobulin G glycome. *Nature chemical biology*, *14*(5), 516-524.

Identified Glycans	Depth %	RT_conflict %	Engine_score %	GlycoViz Composite score	Best MSFragger glycan q-value	Ground Truth
**A2G0F**	0.9673	0.9571	0.0543	0.7806	0	TRUE
**A2G1F**	0.757	0.8381	0.3458	0.7072	0	TRUE
**A2G2F**	0.4673	0.919	0.2393	0.6024	0.1217	TRUE
**A1G0F**	0.4673	0.7286	0.3291	0.5442	0	TRUE
**A1G1M4F/A1G0M5F**	0.1729	0.919	0.2288	0.4825	0	TRUE
**A2G3F/A2G1M5F**	0.1729	0.9857	0.0878	0.481	0.0652	TRUE
**A1G1F/A1G0M4F**	0.3972	0.4048	0.7547	0.4717	0	TRUE
**M5**	0.5327	0.5	0.1876	0.4506	0	TRUE
**A1G1M5F/A1G2M4F**	0.0748	0.9714	0.1074	0.44	0.0824	TRUE
**A3G5F**	0.0561	0.9857	0.0699	0.4307	0.0917	**FALSE**
**A1G1M5/A1G2M4F/A1G2M4**	0.0888	0.919	0.0993	0.423	0	TRUE
**A3G0F**	0.757	0.1762	0.0147	0.3762	0.025	TRUE
**A2G0F2**	0.3972	0.219	0.2533	0.2972	0.2900	**FALSE**
**A3G3**	0.1121	0	0.1059	0.066	0.1217	**FALSE**
**Hex(8)**	0.0374	0.019	0.0629	0.0352	0	**FALSE**
**Hex(9)**	0.0187	0.0095	0.0315	0.0176	0.025	**FALSE**

Supplement Data 3: Algorithm validation performed using NIST monoclonal antibody reference material 8671, leveraging its established glycoform profile as a definitive benchmark. A publicly available NIST mAb LC-MS/MS dataset (MassIVE accession: MSV000093562) was analyzed using the MSFragger-Glyco mode search engine , and subsequently processed with GlycoViz. The presented table summarizes the detailed validation scoring and final composite score. For comparison, the minimum MSFragger glycan q-value for each glycan type is also listed. The ground truth column is true if the glycan type was identified in any of the two published NIST mAb glycan studies in the references below. The table is ranked by the glycoViz composite score. The 5 false positives ranked 10th and 13th to 16th among 16 identified glycans, consistently scoring below true identifications. This stratification yielded a receiver operating characteristic area under the curve (ROC-AUC) of 0.96, demonstrating the algorithm's robust capacity to distinguish true glycan identifications from false positives.

Data source: NIST mAb LC/MS-MS data downloaded from https://massive.ucsd.edu/ProteoSAFe/dataset.jsp?accession=MSV000093562. Reference: Remoroza, C. A., Burke, M. C., Mak, T. D., Sheetlin, S. L., Mirokhin, Y. A., Cooper, B. T., ... & Stein, S. E. (2024). Comparison of N-glycopeptide to Released N-glycan abundances and the influence of glycopeptide mass and charge states on N-linked glycosylation of IgG antibodies. *Journal of proteome research*, *23*(4), 1443-1457.

Ground truth data sources: Table 7 in https://documents.thermofisher.com/TFS-Assets/CMD/Application-Notes/an-21781-lc-ms-ptm-nistmab-rm-8671-an21781-en.pdf
and Table 1 in https://pmc.ncbi.nlm.nih.gov/articles/PMC5680791/ Hilliard, M., Alley Jr, W. R., McManus, C. A., Yu, Y. Q., Hallinan, S., Gebler, J., & Rudd, P. M. (2017, November). Glycan characterization of the NIST RM monoclonal antibody using a total analytical solution: From sample preparation to data analysis. In *MAbs* (Vol. 9, No. 8, pp. 1349-1359). Taylor & Francis.
